# Microwave ablation combined with cementoplasty under real-time temperature monitoring in the treatment of 82 patients with recurrent spinal metastases after radiotherapy

**DOI:** 10.1186/s12891-022-05999-y

**Published:** 2022-11-29

**Authors:** Baohu Wang, Kaixian Zhang, Xusheng Zhang, Sen Yang, Miaomiao Hu, Peishun Li, Wanying Yang, Jing Fan, Chao Xing, Qianqian Yuan

**Affiliations:** Department of Oncology, Tengzhou Central People’s Hospital Affiliated to Jining Medical College, 181 Xingtan Road, Tengzhou, 277500 Shandong Province China

**Keywords:** Microwave ablation, Cementoplasty, Real-time temperature monitoring, Recurrent spinal metastases

## Abstract

**Background:**

The spine is the most frequently affected part of the skeletal system to metastatic tumors. External radiotherapy is considered the first-line standard of care for these patients with spine metastases. Recurrent spinal metastases after radiotherapy cannot be treated with further radiotherapy within a short period of time, making treatment difficult. We aimed to evaluate the effectiveness and safety of MWA combined with cementoplasty in the treatment of spinal metastases after radiotherapy under real-time temperature monitoring.

**Methods:**

In this retrospective study, 82 patients with 115 spinal metastatic lesions were treated with MWA and cementoplasty under real-time temperature monitoring. Changes in visual analog scale (VAS) scores, daily morphine consumption, and Oswestry Disability Index (ODI) scores were noted. A paired Student’s t-test was used to assess these parameters. Complications during the procedure were graded using the CTCAE version 5.0.

**Results:**

Technical success was attained in all patients. The mean VAS score was 6.3 ± 2.0 (range, 4–10) before operation, and remarkable decline was noted in one month (1.7 ± 1.0 [*P* < .001]), three months (1.4 ± 0.8 [*P* < .001]), and six months (1.3 ± 0.8 [*P* < .001]) after the operation. Significant reductions in daily morphine consumption and ODI scores were also observed (*P* < .05). Cement leakage was found in 27.8% (32/115) of lesions, with no obvious associated symptoms.

**Conclusion:**

MWA combined with cementoplasty under real-time temperature monitoring is an effective and safe method for recurrent spinal metastases after radiotherapy.

## Background

The bone is the third most common organ affected by metastases [1], Of which, the spine is the most frequently affected part [2]. Conventional, palliative, and short-course external radiotherapy is considered the first-line standard of care for these patients with spine metastases; however, the complete response rate to pain is low, usually between 10 and 20% [3]. Moreover, almost half of the remaining patients have recurrent pain at a median of 16 weeks following the treatment [4]. Recurrent spinal metastases after radiotherapy cannot be treated with further radiotherapy within a short period of time, making treatment difficult.

Percutaneous ablation techniques have recently gained acceptance; compared to other ablation methods, microwave ablation (MWA) provides several potential advantages [5]. Cementoplasty can enhance bone stability and effectively prevent and treat osteoporosis and pathological fractures [6, 7]. A small number of published studies have evaluated the treatment of spinal metastases treated by MWA combined with cementoplasty [8–11]. However, due to the rapid heating rate of MWA and the large ablation area, there is a potential for damage to the spinal cord and nerves. This makes real-time temperature monitoring during the ablation process very important. Therefore, we aimed to evaluate the effectiveness and safety of MWA combined with cementoplasty in the treatment of spinal metastases after radiotherapy under real-time temperature monitoring.

## Methods

All the patients were not randomized. All the treatments were performed by the same team of operators, with two doctors having 10 years experience. The inclusion criteria were as follows: (1) pathologic evidence of primary cancer; (2) spinal metastasis confirmed by computed tomography (CT) and magnetic resonance imaging (MRI) scan; (3) recurrence of pain after radiotherapy, and (4) pain (visual analog scale [VAS] score ≥ 4) that severely affected the patient’s quality of life.

The exclusion criteria were as follows: (1) coagulation disorder and infections; (2) end-stage malignant tumors with heart and lung failure; or (3) metastases causing symptomatic spinal cord compression.

### Microwave ablation

**Fig. 1:**
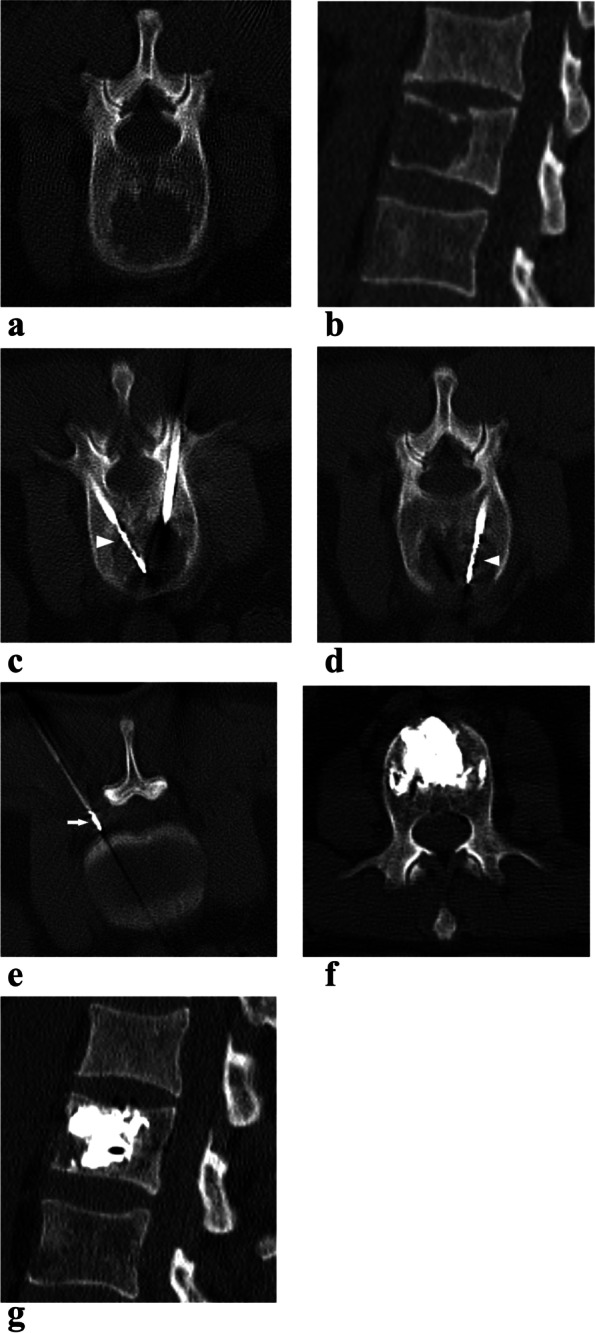
A 58-year-old man with lung adenocarcinoma with L2 osteolytic metastasis (recurrence after radiotherapy) treated with MWA in combination with cementoplasty. L2 osteolytic metastasis showed on axial and sagittal reconstructed CT (**a**, **b**). The microwave ablation antennas (arrowheads) were inserted into the lesion through bilateral access (**c**, **d**). The thermometric electrode (arrow) was inserted into the left intervertebral foramen (**e**). The cement deposited in the vertebral body without significant leakage on axial and sagittal CT (**f**, **g**)

MWA was performed using the ECO-100A1 MW ablation system with a frequency of 2450 ± 10 MHz and an output level of 0 to 100 W (ECO Microwave Electronic Institute, Nanjing, China) under CT (SOMATOM Definition AS; Siemens Healthineers, Erlangen, Germany) guidance. The patients lay prone on the CT flatbed and were fixed with a vacuum negative pressure pad. The lateral position was an alternative if the patient could not lie prone. A positioning grid was placed on the skin surface, and a 5-mm slice CT was performed to choose the puncture path. The surgical area was disinfected using povidone-iodine followed by cleaning with a sterile surgical towel. Conscious sedation of patients was achieved with continuous intravenous infusion of fentanyl citrate (0.1 mg/2 mL), which was diluted 1:10 with saline solution; then, local anesthesia was administered with subcutaneous injection of 1% lidocaine hydrochloride and 0.25% ropivacaine hydrochloride. When 13-gauge bone needles were inserted into the pedicle step-by-step close to the anterior edges of the lesions under imaging guidance, continuous 3D reconstruction during needle insertion was performed to adjust the angle and direction of needle insertion and guide the bone puncture needle accurately into the target area. Subsequently, the cores were pulled out, the antenna was coaxially inserted into the lesion, the bone needle was retrograded to expose the anterior portion of the ablation antenna for 1.5–2.0 cm, and the thermocouple was coaxially inserted in the spinal canal or in the ipsilateral intervertebral foramen to monitor the temperature (Fig. 1). The maximum accepted temperature threshold was 42 ℃ [13, 14]. When the temperature exceeded 42℃, intrathecal carbon dioxide or 5% ice glucose was injected into the spinal canal to protect the spinal cord. According to ablation parameters provided by the manufacturer and the location of the lesion, appropriate power and time were selected empirically. The MWA power was 20–40 W (mean 31.7 ± 5.0 W). Short, repeated microwave cycles (30–90 s) were performed. The total ablation time was 3–5 min (mean, 3.6 ± 1.1 min). Unilateral ablation (20, 17.4%) was performed if the lesions were located on one side without crossing the midline; bilateral ablation (95, 82.6%) was performed if the lesions crossed the midline. Lower-limb clinical examinations (motor and sensory) were performed during the operation, and the patients were asked to inform us about the presence of lower-limb pain or paresthesia.

### Cementoplasty

Several 1-mL syringes filled with polymethylmethacrylate (PMMA) (Heraeus Medical GmbH, Wehrheim, Germany) were kept in ice saline to prolong the setting time. Ten minutes after MWA, the PMMA was slowly injected into the ablative lesion; CT was performed immediately to visualize the flow of PMMA after every 1 mL injection. When the PMMA was close to the posterior edge of the vertebral body, the dose of a single injection was reduced to 0.3–0.5 ml. A single vertebral body was scanned each time, and the scanning time was approximately 3 s. While filling the ablation area with PMMA as much as possible, if the PMMA leaked into the spinal canal and intervertebral foramen, the injection was stopped. The mean volume of bone cement per lesion was 5.7 ± 1.9 mL (range, 2–10 mL). CT was performed after the operation to evaluate adequate filling and leakage of cement.

Technical success was defined as precise placement of the antenna in the lesion, achievement of the target ablation power and time, and placement of an adequate amount of cement in the lesion.

### Follow-up schedule

The follow-up time was at least 6 months. The Oswestry Disability Index (ODI), VAS scores, morphine dosage, side effects, and complications of all patients were recorded through telephonic enquiries or at the outpatient department. CT and MRI scans were performed 3 and 6 months after the operation.

### Outcome assessment

The primary outcomes were VAS scores and morphine dosages, the secondary outcomes were complications. The VAS scores and morphine dosages were used to assess pain. The ODI score was used to assess the degree of disability [15]. Complications during the procedure were graded using the CTCAE version 5.0.

### Statistical analyses

SPSS Statistics version 26 software (IBM Corp, Armonk, NY, USA) was used to perform the analysis. Mean and standard deviation or median and range were used to express the numerical date. A paired Student’s t-test was used to assess the VAS scores, morphine doses, and ODI scores. All tests were two-tailed and a *P* value < 0.05 was considered statistically significant.

## Results

### Clinical data

The local Institutional Review Board (Ethics number: 2020-Ethics Review-12) approved this study. Informed consent was obtained from all patients before treatment. From May 2015 to December 2021, 82 patients with 115 lesions were enrolled. Of these, 48 lesions were located in the thoracic vertebrae and 67 in the lumbar vertebrae. All primary lesions were histopathologically diagnosed, and the spinal metastases were confirmed by a combination of CT and MRI before the operation. All patients had received external irradiation with the scheme of 10 × 3 Gy prior to MWA combined with cementoplasty, of which 53 received concurrent or sequential systemic therapy.The median time after radiotherapy was 10.2 (rang, 5–21) months. According to the modified Shimony, the degree of epidural invasion and spinal cord compression is classified as type A, type B, and type C [12], of which type C was excluded from our study. An interdisciplinary team including interventional radiologists, surgeons, and oncologists evaluated all patients before the operation. The baseline characteristics are listed in Tables 1 and 2.

**Table 1:** Baseline characteristics

	Number	Percent (%)
Age (years)		
Median (range)	63 (18–88)	
Sex		
Male	49	59.8
Female	33	40.2
Number of lesions		
One	57	69.5
Two	18	22
Three	6	7.3
Four	1	1.2
Primary tumor		
Lung	29	35.4
Bladder	1	1.2
Bile duct	1	1.2
Multiple myeloma	1	1.2
Liver	5	6.1
Femur	1	1.2
Colon	2	2.4
Choroidal malignant melanoma	1	1.2
Prostate	3	3.7
Breast	11	13.4
Kidney	5	6.1
Esophagus	7	8.5
Stomach	7	8.5
Lower jaw	1	1.2
Pancreas	1	1.2
Unidentified primary adenocarcinoma	2	2.4
Uterus	2	2.4
Mediastinum	2	2.4

**Table 2:** Baseline characteristics of lesions

	Number	Percent (%)
Type of lesion		
Lytic	88	76.5
Osteogenic	6	5.2
Mixed	21	18.3
Intact posterior edge		
Yes	67	58.3
No	48	41.7
The degree of epidural invasion	48	41.7
Type A	32	27.8
Type B	16	13.9
Compression fracture		
Yes	46	40
No	69	60

MWA combined with cementoplasty under real-time temperature monitoring was successfully performed in all patients, and all patients were followed up for at least 6 months.

### Pain relief

**Fig. 2:**
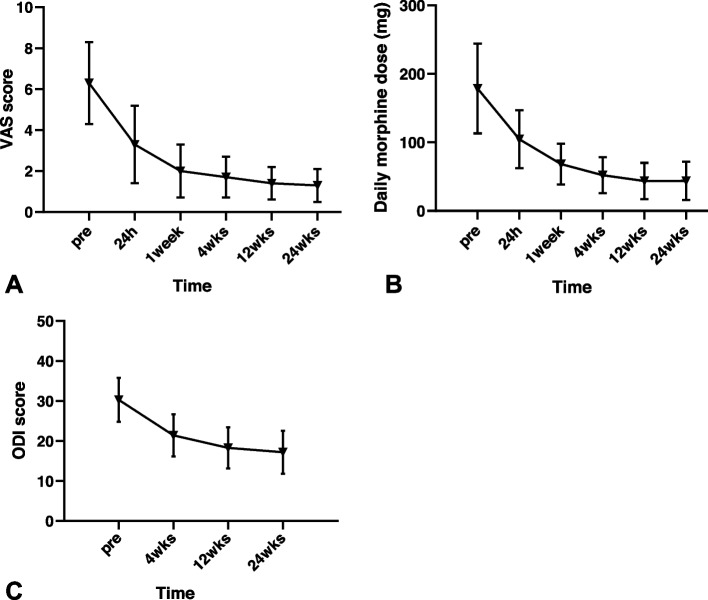
The changes of visual analog scale (VAS) scores (**A**), Daily morphine dose (mg) (**B**), and Oswestry Disability Index (ODI) (**C**) after operation

The preoperative VAS score was 6.3 ± 2.0 (range, 4–10); the postoperative VAS score decreased in 24 h (3.3 ± 1.9, *P* < 0.05), one week (2.0 ± 1.3, *P* < 0.001), four weeks (1.7 ± 1.0, *P* < 0.001), 12 weeks (1.4 ± 0.8, *P* < 0.001), and 24 weeks (1.3 ± 0.8, *P* < 0.001) (Fig. 2A). Compared to the preoperative morphine dose (178.5 ± 65.7 mg; range, 80–300 mg), the postoperative morphine doses decreased significantly in 24 h (104.5 ± 42.3 mg, *P* < 0.05), one week (68.4 ± 29.7 mg, *P* < 0.001), four weeks (52.0 ± 26.4 mg, *P* < 0.001), 12 weeks (43.5 ± 26.7 mg, *P* < 0.001) and 24 weeks (43.9 ± 28.1 mg, *P* < 0.001) (Fig. 2B). During the follow-up, eight patients experienced recurrence in pain, of which five underwent systemic treatment, three underwent pain management with morphine.

### Degree of disability

Compared to preoperative values (30.3 ± 5.5; range, 20–45), the postoperative ODI score decreased in four weeks (21.4 ± 5.3, *P* < 0.005), 12 weeks (18.3 ± 5.2, *P* < 0.001), and 24 weeks (17.2 ± 5.4, *P* < 0.001) (Fig. 2C).

### Complications

Asymptomatic cement leakage (one degree) was found in 32/115 (27.8%) lesions in our study. Forty sites of cement leakage were identified, of which nine (22.5%) were in the vertebral canal, five (12.5%) were in the posterior vertebral vein, 18 (45%) were in the intervertebral disc, and eight (20%) were in the paraspinal region. However, the cement leakages showed no obvious relationship with the presence of an intact posterior edge of the spine (Table 3). Other complications, such as hematoma, skin burns, bone cement embolism, infection and periprocedural death, were not observed in our study.

**Table 3:** Relationship between spine posterior edge and bone cement leakage

Intact posterior edge	Total (n)	Bone cement leakage		χ^2^ test	
		Yes, n (%)	No, n (%)	χ^2^ value	*P* value
Yes	67	18 (26.9)	49 (73.1)	0.074	0.786
No	48	14 (29.2)	34 (70.8)		

## Discussion

Currently, painful spinal metastases are treated to relieve pain and improve the quality of life. Several effective mini-invasive treatments for the management of spinal metastases are currently available, including embolization, thermal ablation, electrochemotherapy, cementoplasty, and MRI-guided high-intensity focused ultrasound [16]. Of which, thermal ablation performed to relieve pain could cause coagulative necrosis of the tumor. Microwave and radiofrequency are commonly used for thermal ablation; compared with radiofrequency, microwave can provide faster heating and target larger ablation areas without grounding electrodes [17]. Microwave is more insensitive to the high impedance of bone desiccation than is radiofrequency, causing deeper thermal penetration, which efficiently heats the bone [15]. However, microwaves cannot improve the biomechanical stability of the bone. Cementoplasty can relieve pain and increase the biomechanical stability of the bone. Combining MWA and cementoplasty can lead to optimal cement distribution and bone stabilization [18, 19]. However, compared to MW-ablation alone, cementoplasty combined with MW-ablation increases the operation time, cost and the risk of complications.

Pusceddu et al. [19] reported that 35 patients with 37 osseous metastases treated by MWA achieved significant pain relief in one week, one month, and six months, without local tumor progression. Another retrospective study showed that the postoperative VAS scores of 69 patients with spinal metastases treated by MWA and cementoplasty decreased significantly at 2–4 weeks (2 ± 1.6) and 20–24 weeks (2 ± 2.1), compared to the preoperative VAS scores (7.0 ± 1.8). No local progression was found in 59/61 patients [9]. Wu L et al. [18] reported 33 high thoracic vertebral metastases treated by MWA and cementoplasty. The mean VAS score, morphine consumption doses, and ODI significantly decreased at 24 h and one, four, 12, and 24 weeks postoperatively, with no local tumor progression during the 24-week follow-up. The results are in accordance with previously reported studies.

Whether cementoplasty can be used in patients with an incomplete posterior wall is still controversial. Most authors consider that neurologic symptoms associated with nerve root- or spinal cord compression are contraindications to cementoplasty [20–22]. However, few studies have demonstrated the feasibility of cementoplasty. Researchers believe that epidural involvement with intractable pain, few other treatment options, and a short life expectancy should not be a contraindication to cementoplasty. Moreover, the patients must be properly screened, and cementoplasty should be performed with conscious sedation [12, 23, 24]. Halpin et al. [25] showed that thermal ablation before cementoplasty could decrease the risk of cement extravasation due to thrombosis of the venous plexus caused by ablation. We found no statistically significant difference in the incidence of cement leakage between groups with incomplete and intact posterior margins. We also found no spinal nerve injury in either group after surgery. Therefore, we believe that incomplete posterior margins should not be a contraindication for performing cementoplasty.

Thermal ablation has the intrinsic risk of nerve damage, which is a serious potential complication of the operation. Several aspects affect the extent and severity of nerve damage such as type of nerve fiber, distance from margins of the ablation zone, presence or absence of intact vertebral cortex, duration of thermal effect, and absolute temperature [26]. The threshold of irreversible nerve damage is 42.2 ℃ for 50–60 min or 70 ℃ for 5 min [27]. Studies have shown that the incidence of nerve injury was 1.4–17.4% [9, 28, 29]. Several methods are used to prevent thermal injury, including active warming/cooling with saline injection, passive insulation with CO_2_ insufflation [30, 31], and continuous temperature monitoring [30, 32, 33]. Additionally, repeated and short ablation cycles to control the diffusion of the ablation zone might be suitable for tumors close to vital structures [10]. In our study, thermocouples were positioned close to vital neural elements for continuous temperature monitoring, and repetitive, short ablation cycles were also used to ensure the safety of thermal ablation.

This study had some limitations. We did not include a control group. Additionally, the study was conducted at a single center, was retrospective in nature, and had a short follow-up duration. Thus, the findings outlining the benefits of MWA combined with cementoplasty need to be confirmed in further studies, and multicenter randomized controlled studies are needed to validate these results and obtain more conclusive findings.

MWA combined with cementoplasty results in reduced VAS and ODI scores, without serious complications, and is a safe and effective method for treating painful recurrent spinal metastases after radiotherapy.

## Data Availability

The datasets used and/or analysed during the current study are available from the corresponding author on reasonable request.
